# Effective Long-Distance Pollen Dispersal in *Centaurea jacea*

**DOI:** 10.1371/journal.pone.0006751

**Published:** 2009-08-25

**Authors:** Matthias Albrecht, Peter Duelli, Martin K. Obrist, David Kleijn, Bernhard Schmid

**Affiliations:** 1 Institute of Environmental Sciences, University of Zurich, Zurich, Switzerland; 2 Biodiversity and Conservation Biology, Swiss Federal Institute for Forest, Snow and Landscape Research WSL, Birmensdorf, Switzerland; 3 Centre for Ecosystem Studies, Alterra, Wageningen, The Netherlands; Trinity College Dublin, Ireland

## Abstract

**Background:**

Agri-environment schemes play an increasingly important role for the conservation of rare plants in intensively managed agricultural landscapes. However, little is known about their effects on gene flow via pollen dispersal between populations of these species.

**Methodology/Principal Findings:**

In a 2-year experiment, we observed effective pollen dispersal from source populations of *Centaurea jacea* in restored meadows, the most widespread Swiss agri-environment scheme, to potted plants in adjacent intensively managed meadows without other individuals of this species. Potted plants were put in replicated source populations at 25, 50, 100 m and where possible 200 m distance from these source populations. Pollen transfer among isolated plants was prevented by temporary bagging, such that only one isolated plant was accessible for flower visitors at any one time. Because *C. jacea* is self-incompatible, seed set in single-plant isolates indicated insect mediated effective pollen dispersal from the source population. Seed set was higher in source populations (35.7±4.4) than in isolates (4.8±1.0). Seed set declined from 18.9% of that in source populations at a distance of 25 m to 7.4% at 200 m. At a distance of 200 m seed set was still significantly higher in selfed plants, indicating long-distance effective pollen dispersal up to 200 m. Analyses of covariance suggested that bees contributed more than flies to this long-distance pollen dispersal. We found evidence that pollen dispersal to single-plant isolates was positively affected by the diversity and flower abundance of neighboring plant species in the intensively managed meadow. Furthermore, the decline of the dispersal was less steep when the source population of *C. jacea* was large.

**Conclusions:**

We conclude that insect pollinators can effectively transfer pollen from source populations of *C. jacea* over at least 200 m, even when “recipient populations” consisted of single-plant isolates, suggesting that gene flow by pollen over this distance is very likely. Source population size and flowering environment surrounding recipient plants appear to be important factors affecting pollen dispersal in *C. jacea*. It is conceivable that most insect-pollinated plants in a network of restored sites within intensively managed grassland can form metapopulations, if distances between sites are of similar magnitude as tested here.

## Introduction

Habitat fragmentation has been recognized as a major threat to the survival of local populations of plants and animals [1], [2]. Fragmentation and isolation can reduce the reproductive success of plants in local populations through reduced availability of suitable mates, loss of genetic variation and inbreeding depression [3]–[7]. In insect-pollinated plants, pollen dispersal is generally the main component of gene flow [8], [9]. This pollen-mediated gene flow may be largely driven by ecological factors that determine the availability and foraging behaviour of the involved pollinators [10], [11]. These factors may include the “direct” effects of life-history traits or taxon-specific foraging behaviour of the pollinators [12], but also effects of habitat variables that “indirectly” affect pollen dispersal by altering the availability and foraging behaviour of pollinators, such as the physical distance between plant patches [e.g. 10], [13], [14], plant population size and density [7], [11], [15] and the surrounding flowering environment, including conspecific and heterospecific co-flowering plants [13], [16], [17]. For example, large source populations may be associated with more pollen effectively transferred over larger distances [10]. A higher abundance and diversity of heterospecific co-flowering plants may attract higher numbers of pollinators, thereby facilitating pollination and seed set of the focal plant species [18], [19]. However, a diverse floral neighborhood may also reduce conspecific pollen deposition by driving potential pollinators away or through increased heterospecific pollen deposition [e.g. 11], [20]. The relative importance of these factors driving effective pollen dispersal (i.e. pollen dispersal with subsequent pollen deposition, fertilization and seed production on a receiving plant) is still poorly understood [10], [11], [21].

In the intensively managed agricultural landscapes of the Swiss lowland, as in many other regions of Central Europe, many plant species are confined to small semi-natural habitat fragments, which are often part of agri-environment schemes [12]–[24]. Agri-environment schemes are financial incentives compensating farmers who apply management prescriptions to promote biodiversity and to improve the general status of the environment [25]. In 2004, such restored habitats covered 13% of the cultivated area of Switzerland [26]. Restored meadows, created by sowing of species-rich seed mixtures and maintained by postponed mowing and no fertilizer application, currently represent by far the most widely adopted agri-environment scheme with a share of approximately 80% [27]. However, agri-environment schemes in intensively managed farmland sometimes fail to significantly promote biodiversity [28]. According to the results of an evaluation of Swiss agri-environment schemes, 51–87% of all restored meadows (depending on biogeographical region) currently do not meet the quality criteria of traditional hay meadows and harbour only few plant species of the Swiss Red List [26]. One possible explanation for these results is that the isolated plant populations in restored meadows suffer from reduced genetic variation and inbreeding depression [e.g. 4], [29]. Knowledge about effective pollen dispersal in insect-pollinated plant species restricted to less intensively managed habitats and the ecological factors determining this insect-mediated pollen dispersal is crucial to improve the spatial design of agri-environment schemes.

In the present study we examined the effects of isolation distance from source populations of *Centaurea jacea* L. in restored meadows on pollinator activity and effective pollen dispersal to single-plant isolates placed into adjacent intensively managed meadows. Since habitat fragmentation usually not only results in increased isolation, but also alters other factors influencing effective pollen dispersal, such as patch size or density, only an experimental approach can disentangle these factors. We used the relatively common *C. jacea* as a model plant species because it generally does not occur in intensively managed meadows and is self-incompatible. This allowed us to directly measure seed set as a function of distance from the source population because seed set was the product of effectively transferred pollen from the source population in the restored habitat [30]. Differential resource limitation, another possible confounding factor influencing seed set was experimentally excluded by using the same soil for all potted plants. In contrast to measurements of the deposition of pollen or pollen analogues on stigmas, our approach allowed us to directly measure gene flow by pollen.

We addressed the following questions: (1) Is there effective pollen dispersal from source populations of *C. jacea* in restored meadows to single-plant isolates in adjacent intensively managed meadows and (2) how does distance from the source population affect this dispersal of pollen? (3) Which pollinator groups are responsible for effective pollen dispersal in *C. jacea*? (4) How does source population size and the flower abundance and diversity of co-flowering plant species influence effective pollen dispersal?

## Materials and Methods

### Study species

*Centaurea jacea* L. sensu stricto (Asteraceae) is a short-lived perennial herb native to Europe. It is typically found in meadows of the Swiss lowland where flowering lasts from early June to mid October. A capitulum contains on average 54.7±1.9 central hermaphroditic disc flowers (n = 10) and a ring of sterile ray flowers. *Centaurea jacea* is insect-pollinated and several studies have demonstrated that it is almost completely self-incompatible [31], [32]. To determine the number of seeds per capitulum produced by selfing in our experiment, we bagged several capitula of every potted plant. The mean seed set of the bagged capitula was 0.31±0.11 seeds per capitulum (n = 128).

### Study sites and experimental design

The experiment was conducted from June to September in 2004 and 2005 in the south-eastern part of canton Aargau near Zurich. The region represents the typical intensively managed agricultural landscape of the Swiss lowland, consisting of a small-scale mosaic of grassland, arable fields and forests.

In 2004, we selected at eleven sites one meadow managed according to the prescriptions of the Swiss agri-environment scheme for the category “restored meadows” and adjacent intensively managed meadows. These restored meadows ranged in area from 0.48 ha to 2.15 ha (mean = 1.05±0.13 ha). Management prescriptions for restored meadows included a postponed first cut of the meadow and no fertilizer or pesticide application. In 2005, the experiment was repeated at eight of the eleven sites selected in 2004. In each of the selected restored meadows there was a population of *C. jacea* with population size ranging from 15 to approximately 160 individuals (mean = 67.2±15.8). No other individuals of *C. jacea* occurred in the adjacent intensively managed meadows within a distance of at least 500 m. In four restored meadows, the populations of *C. jacea* had existed already before the meadows were managed according to the prescriptions of the scheme. At the other seven sites, *C. jacea* had been sown by the farmer, together with other plants forming part of a standardized seed mixture to enhance plant species richness in the restored habitat. In all restored meadows, populations of *C. jacea* had existed for at least 6 years at the time the experiment was conducted.

At each study site, one potted plant of *C. jacea* was placed in the restored meadow (control) at a distance of about 1 m from the nearest conspecific individual. In the adjacent intensively managed meadows, one potted plant was placed at each of the three distances 25, 50 and 100 m from the source population at each site (n = 11) and at four sites also at a distance of 200 m (n = 4). In order to prevent pollen transfer between potted plants within the intensively managed meadows (hereafter referred to as “single-plant isolates” or “isolates”), all but one of them at each site were bagged and the bags opened such that only one isolated plant in the intensively managed meadows was accessible for pollinators at any one point in time. Flowers of isolates were exposed to pollinators for a total of approximately 3 consecutive weeks. The time in the flowering season a potted plant at a certain distance class was exposed to pollinators was varied over the 11 study sites at random. Because *C. jacea* is self-incompatible [31], [32], seed set can only occur after successful pollen transfer from the source population to the single plant isolate. In each replicate the access to pollinators was granted in a different sequence to prevent confounding effects among distance and time-dependent covariables.

### Seed set

Single-plant isolates of *C. jacea* were regularly checked throughout the seasons of 2004 and 2005. When all flowers had wilted, the capitula of *C. jacea* were bagged with fine-mesh gauze to prevent loss of ripe seeds between recordings. Within the subsequent two weeks, bagged capitula were collected and stored in the laboratory. On average, a potted plant with access to pollinators had 3.0±0.2 open capitula. In total, 245 open pollinated capitula were analysed for seed set, 174 in 2004 and 71 in 2005. Seed set was defined as the total number of fully developed seeds per capitulum.

In only 2.1% of all seeds, signs of herbivory (i.e. seeds with holes or larvae) were detected. We did not observe any patterns in seed predation rate (number of seeds with signs of herbivory per total number of seeds of a capitulum) with distance from restored meadows (log(distance) contrast: *F*_1,13_ = 0.24, *P* = 0.634; see “Statistical analysis” section below) making it unlikely that pre-dispersal seed predation affected our results.

### Flower visiting insects

On each potted plant of *C. jacea*, flower-visiting insects were surveyed three times between 1 June and 14 September 2004. During 20 minutes, each flower-visiting insect was captured with a fine-meshed butterfly net. Sampling was carried out between 10 am and 4 pm in suitable weather conditions (sunshine and low wind velocity). Time of day was varied for the sampling of the same potted plant. All collected specimens of the three most important taxa, bees (Hymenoptera: Apoidea), hover flies (Diptera: Syrphidae) and butterflies (Lepidoptera), were identified to species level. As we hypothesized that body size affects a flower visitors's foraging range and hence its potential contribution to effective pollen dispersal, each identified species was assigned to a size class. The classification of bees and hover flies was based on that of Schweiger *et al.* [33], but the two classes “small” and “very small” [33] were merged into one “small” size class and the classes “large” and “medium” were merged into a single “large” size class to have sufficiently high numbers in each class for statistical analyses. Each butterfly species was assigned to the “large” size class. Due to low numbers of species in small bees, large bees, small hover flies, large hover flies and butterflies and low numbers of individuals of butterflies and small bees, we report only the results of the analyses of pooled data of the number of species and individuals of bees, hover flies, large-sized visitors and small-sized visitors in the following. The patterns revealed by analyses on separate functional groups and species groups were consistent with those on the pooled data.

### Vegetation survey

In 2004, species richness and flower abundance of the flowering plants surrounding the potted *C. jacea* were recorded to examine their influence on species richness and abundance of pollinators visiting the experimental plants and thereby potentially affecting the effective pollen dispersal. Five 2×5 m plots spaced 2.5 m apart were arranged in a linear fashion on both sides of each potted plant, parallel to the border between the restored and intensively managed habitat. Flowering plant abundance was estimated as the percentage of the plot area covered by vegetative and flowering parts of flowering individuals of all five plots and mean percentage cover was calculated. The estimates were recorded during three survey rounds from the beginning of June to the end of August.

### Statistical analysis

To test for a significant effect of distance on the seed set per capitulum, a generalized linear model with quasi-Poisson errors was used. To account for overdispersion in the data quasi-*F* tests were performed by calculating ratios of mean deviance changes [34]. Site and potted plant within site as blocking factors and distance as treatment factor were fitted to the pooled data set of both years. The deviance for the distance factor was decomposed into a contrast control vs. single-plant isolates (plants isolated by 25, 50,100 and 200 m), and the latter into a log(distance) contrast and the remaining deviation from log-linearity. Between-year variation was tested by including year as a treatment factor in the model. To examine whether herbivory rate (arcsine square-root transformed) was related to distance from source population a linear model was fitted using the same model structure as described above.

In order to test whether open pollinated capitula of the most isolated *C. jacea* plants at a distance of 200 m from source populations were more likely to set seeds than bagged capitula, we fitted a logistic model with the treatment factor bagging (bagged or open pollinated) nested within year, and year nested within site, to the binary response variable seed set (≥1 seeds set per capitulum) or no seed set (0 seeds set per capitulum). A logistic model was used because most bagged capitula did not set any seeds. Quasi-binomial errors and *F* tests were used to account for overdispersion in the data [34]. Only plants isolated by 200 m from the source populations were considered in this analysis.

To examine the effect of the habitat variables source population size, plant species richness and flowering plant abundance in the restored meadow and plant species richness and flowering plant abundance in the intensively managed meadow surrounding the single-plant isolates and pollinator variables taxon and body size class on seed set of *C. jacea*, all these variables were separately tested as covariables in the generalized linear model described above. These analyses were restricted to the single-plant isolates in intensively managed meadows. Because the covariables were measured in 2004, only the dataset of the seed set of 2004 was considered for the analyses of covariance. Main effects of the covariables measured at the site levels (source population size, species richness of flowering plants in restored meadows and flower abundance in restored meadows) were tested at the site level, whereas all other covariables were tested at the single-plant isolate level.

All statistical analyses were done in R 2.3.1 (R Development Core Team 2006). Arithmetic means±1 standard error are reported.

## Results

### Seed set as a function of distance from pollen donors

**Figure 1:**
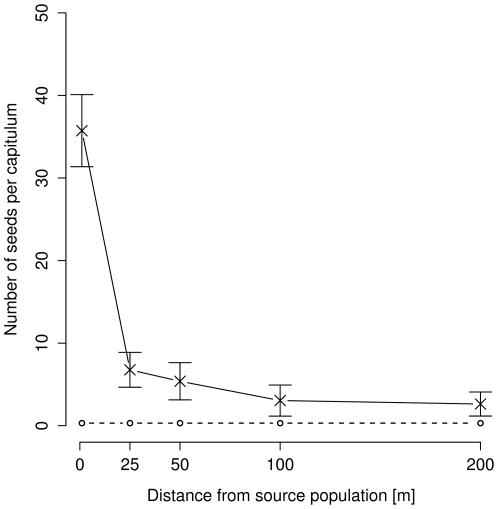
Seed set of *Centaurea jacea* in source populations and single-plant isolates. Seed set (mean±1 SE) indicates effective pollen dispersal from source populations in restored meadows (0 m) to single-plant isolates at distances of 25, 50, 100 m (all n = 11), and 200 m (n = 4) from the sources in adjacent intensively managed meadows. The dashed line marks mean seed set of bagged capitula (n = 128). Pooled data of the seasons 2004 and 2005 are shown.

The mean number of seeds per capitulum (seed set) of *C. jacea* was significantly higher within the source populations in restored meadows (35.7±4.4) than outside the source populations in intensively managed meadows (4.8±1.0) (*F_1,33_* = 91.72, *P*<0.001). The single-plant isolates in the intensively managed meadows set fewer seeds per capitulum the further away they were from the source population (log(distance) contrast: *F_1,33_* = 7.38, *P* = 0.010). Seed set declined from 6.8 to 2.6 seeds per capitulum at 25 m and 200 m (Fig. 1). This represented 18.9% and 7.4% of the value of the control in the restored habitat. The probability of capitula of the most isolated plants at a distance of 200 m with access to flower visitors to set seeds was significantly higher than that of bagged capitula of the same potted plants (*F_1,23_* = 50.35, *P*<0.001), indicating effective pollen dispersal over this distance. However, 42% of plants isolated by 200 m from source populations set no seeds, indicating relatively high spatio-temporal variability in pollen dispersal. Seed set per capitulum was higher in 2005 than in 2004 (*F_1,33_* = 6.21, *P* = 0.018).

### Ecological factors affecting effective pollen dispersal to isolates

In 48 hours we collected 425 flower-visiting insects on flower heads of *C. jacea*. Hover flies and bees were the most abundant visitor groups and the species richness of both visitor groups was similar (Table 1).

**Table 1:** Insects visiting potted plants of *Centaurea jacea*.

Pollinator group	Number of individuals	Number of species
Bees	162	19
Small bees	27	11
Large bees	135	8
Hover flies	192	18
Small hover flies	84	13
Large hover flies	108	5
Other flies	46	–
Butterflies	25	7
Total	425	

Flower-visiting insects were captured in 2004 during 48 hours of sample time. Bees (Hymenoptera: Apidae), hover flies (Diptera: Syrphidae) and butterflies (Lepidoptera: Diurna) were identified to species level. For the classification of pollinators into large-sized and small-sized groups see “Materials and Methods” section.

**Figure 2:**
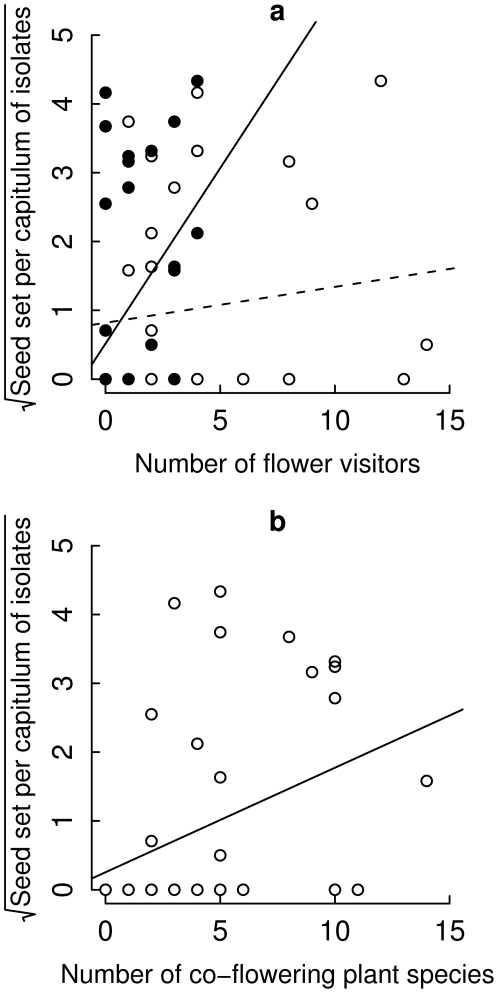
Relationship between covariables and seed set of *Centaurea jacea* isolates. Relationship between (a) the number of individuals of bees (filled circles) and flies (open circles) sampled during 60 min and seed set of single-plant isolates of *C. jacea* (square-root transformed) and (b) source population size and seed set of single-plant isolates (square-root transformed). Because covariables were sampled only in 2004, only the seed set data of 2004 was used for the analyses of covariance. Note that simple linear regressions are shown, while the results reported in Table 2 are obtained from a more complex model using quasi-Poisson errors (see “Statistical analysis” section).

Analyses of covariance of the data from 2004 showed significant positive relationships between the number of both, large and small flower visitors, and seed set (Table 2). Whereas species richness of large flower visitors was significantly positively related to seed set of isolates, there was a trend for a positive relationship among small flower visitors and seed set (Table 2). We found a negative interaction with log(distance) for large but not small flower visitors, indicating a flatter slope in the pollen dispersal function if isolates were visited by higher numbers of large flower visitors. Furthermore, there were significant positive relationships between the number of individuals or species of bees and seed set (Table 2; Fig. 2). In contrast, no significant relationship was found between the number of individuals of flies and seed set of *C. jacea* (Table 2; Fig. 2). The number of species of bees and individuals of flies showed negative interactions with log(distance) (Table 2), indicating flatter slopes in the relationship of seed set and log(distance) with higher numbers of these flower visitors. The number of bee individuals also showed a trend for a negative interaction with log(distance) (Table 2).

**Table 2:** Covariables influencing effective pollen dispersal from source populations to single-plant isolates of *Centaurea jacea*.

		Deviance change (%)	Main effect of covariable			Deviance change (%)	Log(distance)			Deviance change (%)	Covariable x log(distance) interaction	
Covariables			*F*	*P*			*F*	*P*			*F*	*P*
**Pollinator species richness**												
Bees	+	11.36	8.22	**0.004**	−	10.92	7.90	**0.005**	−	6.47	4.68	**0.031**
Small-sized pollinators	+	3.72	2.74	**0.098**	−	18.23	13.63	**<0.001**		2.84	2.09	0.148
Large-sized pollinators	+	14.28	13.65	**<0.001**	−	16.41	15.69	**<0.001**	−	7.57	7.24	**<0.001**
**Pollinator abundance**												
Bees	+	11.87	7.70	**0.006**	−	11.23	7.28	**0.007**	−	4.42	2.87	**0.090**
Flies		2.25	2.50	0.114	−	18.60	20.64	**<0.001**	−	10.79	11.98	**0.001**
Small-sized pollinators	+	9.26	7.03	**0.008**	−	15.22	11.54	**0.001**		3.23	2.45	0.118
Large-sized pollinators	+	11.90	11.81	**0.001**		13.48	13.38	**0.001**	−	4.71	4.67	**0.031**
**Habitat variables**												
Source population size of *C. jacea*		8.43	2.59	0.142	−	22.07	17.08	**<0.001**	−	12.40	9.59	**0.002**
Plant species richness restored meadows		2.04	0.51	0.491	−	22.07	13.61	**<0.001**		2.41	1.49	0.223
Plant species richness intensively managed meadows	+	7.96	5.58	**0.018**	−	27.41	19.21	**<0.001**		0.01	0.09	0.761
Flower abundance restored meadows		1.91	0.48	0.507	−	22.07	11.89	**0.001**		0.00	0.01	0.969
Flower abundance intensively managed meadows	+	3.67	3.12	**0.077**	−	25.59	21.76	**<0.001**		0.01	0.08	0.780

Summary of generalized linear model analyses using quasi-Poisson errors and F-tests to explore the effects of covariables (number of species and number of individuals of flower visitor groups visiting potted plants of *C. jacea* during 60 min and habitat variables) on mean seed set of single-plant isolates (main effect of covariable) and the slope of the decline of this seed set (interaction of covariable with log(distance) from source population). Source populations were restricted to restored meadows and single-plant isolates were set up in surrounding intensively managed meadows at 25, 50, 100 and 200 m distance from source populations. A plus (+) indicates a positive effect, a minus (–) a negative effect. A positive effect of a covariable on the mean seed set of single-plant isolates indicates that more pollen was effectively transferred from source populations to single-plant isolates. A negative covariable x log(distance) interaction indicates a less steep decline of effectively transferred pollen with distance from source populations. In all analyses, the mean seed set in 2004 was used as the dependent variable, since the covariables were sampled in 2004 only. Main effects of the covariables population size, species richness of flowering plants in restored meadows and flower abundance in restored meadows were tested at the site level, all other covariables were tested at the single-plant isolate level. The fitted models are described in the “Statistical analysis” section.

Species richness and flower abundance of plants surrounding the single-plant isolates in the intensively managed meadows showed a positive relationship or a strong trend for a positive relationship, respectively, with seed set of *C. jacea* isolates (Table 2; Fig. 2). However, seed set of isolated *C. jacea* plants in intensively managed meadows was not significantly affected by plant species richness or abundance of co-flowering plants in the restored meadows where the pollen donors grew (Table 2).

The size of the source population size of *C. jacea* significantly affected the slope of the relationship between the mean seed set and the log(distance) from the source (Table 2). Source population size increased effective pollen dispersal over the larger distances, thus leading to flatter slopes.

## Discussion

### Effective pollen dispersal as a function of distance from the source population

The proportion of capitula of *Centaurea jacea* plants with access to pollinators that set seeds at a distance of 200 m was significantly higher than that produced only by selfing. This suggests effective long-distance pollen dispersal by insect pollinators from source populations of *Centaurea jacea* in restored meadows to single plant isolates at least up to 200 m. However, 42% of open pollinated plants isolated by 200 m from source populations set no seeds. Thus, at some sites these single-plant isolates did not receive pollen from the source populations, at least in one of the two years studied, with generally higher levels of seed set in 2005. These findings are in line with many other studies documenting high spatial and temporal variability in pollinator populations and pollination services, which can result in a high variation in the reproductive success of animal-pollinated plants [35,36 and references therein].

The decline in the seed set of *C. jacea* was most pronounced over the first 25 m, with relatively little decline between 100 m and 200 m distance from source populations. This suggests that the precise curve for pollen dispersal may be somewhat more fat-tailed [e.g. 37] than described by the exponential decay assumed with the log(distance) contrast used here. A fat-tailed pollen dispersal curve with distance from a source population is predicted by the changing pollen carryover model of Morris et al. [38], proposing a systematic increase in the pollen carryover fraction during a sequence of flower visits by a pollinator.

Most previous studies focused on pollen dispersal in dense stands of a plant species and report short-distance pollinator flights to neighboring conspecifics and pollen dispersal restricted to a few meters, as predicted by optimal foraging theory [39]–[42]. In a metapopulation context, however, a pollinator is expected to “skip” to the next patch of conspecific plants if it reaches the edge of the current population, depending on the degree of specialization of the pollinator (fixed or short-term due to flower constancy) [21]. Hence, effective pollen dispersal in such a context is expected to occur over much longer distances, as confirmed by this study. Few studies directly investigated pollen dispersal from source populations of other natural, insect-pollinated plant species at relatively large spatial scales [10], [13], [17], [21]. Similarly to our results, Duncan et al. [17] found a significant decline of conspecific outcross pollen receipt in synthetic populations of six flowers of *Dianella revoluta* up to approximately 50 m distance from source populations in nature reserves, with little further decline between 50 m and 400 m. Kwak et al. [13] counted only a few conspecific pollen grains on emasculated flowers of *Scabiosa columbaria* isolated by 200 m from a source population. However, Schulke and Waser [21] found that mean seed set of emasculated flowers of *Delphinium nuttallianum* in isolates consisting of 16 plants declined slowly with increasing distance from source populations up to 400 m, with an average fruit set of 69.8% at isolates. How pollinator and habitat variables may possibly cause such differential levels of pollen dispersal is discussed in the next section.

Using single-plant isolates, our measurements probably provide minimum estimates of effective pollen dispersal from source populations to small, isolated populations. An increase in the size of the “recipient“ populations is generally associated with a higher rate of pollen flow from the source population [7], [10], [43], even though the relative proportion of immigrant pollen of the total pollen pool usually decreases with an increase of the “recipient” population size [10], [13], [43]. Thus, the level of pollen dispersal from source populations to large, isolated populations is likely to be higher than that reported here for single-plant isolates, although we cannot assess levels of such pollen flow. Nevertheless, our finding that even a majority of single-plant isolates at a distance of 200 m received pollen from source populations implies genetic connectedness of most populations of *C. jacea* separated by less than 200 m.

Very small populations have been reported to be even totally overlooked by pollinators [10], [44]. In our experiment, however, most *C. jacea* plants were apparently sufficiently attractive to flower visitors to be pollinated even when isolated by large distances (100–200 m) from the nearest source population. Indeed, *C. jacea* has a relatively large floral display, a trait documented to increase the attractiveness for pollinators [reviewed in 45]. Furthermore, *C. jacea* is an important nectar plant for both butterflies and social bees [46] and was visited more frequently by social bees and butterflies than most other co-flowering plant species at the sites examined in this study (pers. obs.). Finally, co-flowering other plant species surrounding the single plants of *C. jacea* further increased effective pollen dispersal to these individuals (see discussion below). For less attractive plant species, effective pollen dispersal may be more spatially restricted. An open question of this and most other experimental studies on effective pollen dispersal is how exactly the observed increase in seed set affects the long-term persistence of isolated populations.

### Ecological factors affecting effective pollen dispersal

The role of ecological factors affecting long-distance effective pollen dispersal is still poorly understood [10], [12], [21], [47]. The results of this experiment indicated that both flower visitor and habitat variables significantly affected long-distance effective pollen dispersal. Bees and flies were the most abundant flower visitors of potted *C. jacea* plants. Our analysis suggested that bees, although less numerous visitors than flies, may have been relatively more important in effective pollen transfer from source to isolated populations of *C. jacea* than flies. Bees, particularly bumblebees and honeybees, are known to forage at spatial scales of several hundreds of meters and generally show a high flower fidelity [e.g. 48]. Large flower visitors usually forage over larger distances than small ones [49] and are therefore expected to be relatively more important for pollen dispersal over large distances. Flower visitor body size did not appear to greatly affect the overall level of pollen dispersal to *C. jacea* isolates in this study. However, we found some evidence that the slope in the decline of effectively transferred pollen with distance from source populations was less steep if plants were visited by higher numbers of individuals and species of large flower visitors. Such an effect was not found for small flower visitors. This finding is an indication that large flower visitors were probably relatively more involved in pollen dispersal over larger distances than small ones. These conclusions, however, are correlative and we did not quantify the amount of pollen insects carried on their bodies when arriving at the single-plant isolates.

Moreover, our findings suggested that pollinators were more likely to visit single-plant isolates of *C. jacea* if they were surrounded by a higher abundance and diversity of co-flowering plant species in the intensively managed meadows. It is conceivable that larger or more diverse collective floral displays of diverse and flower-rich intensively managed meadows attracted more foraging pollinators from restored meadows, thereby facilitating seed set of isolated *C. jacea* plants [e.g. 11], [18], [19]. However, such potentially facilitative effects of the floral neighborhood might be limited to a constrained set of circumstances, e.g. when plants occur at very low population densities or as single isolated plants [18], [19], as it was the case in our study. In contrast, Schulke and Waser [21] hypothesized that their finding of generalist queen bumblebees and hummingbirds dispersing pollen of *Delphinium nuttallianum* to experimental isolates over larger distances (≥400 m) and in higher quantities than reported for previous studies may be partly explained by the fact that only few heterospecific plant species flowered during the experiment, since pollinators may have skipped long distances with few co-flowering species to fly to isolates. However, Kwak et al. [50] found that bumblebees did not move among populations of *Phyteuma nigrum* separated by 150 m, and suggested that such movement might have been promoted by the presence of intervening, co-flowering heterospecific plants. The question under what circumstances a diverse, flower-rich environment can promote or reduce long-distance pollen dispersal requires further investigation.

Finally, we found that larger source populations resulted in a less steep slope of the pollen dispersal function and accordingly to a more effective pollen dispersal to remote single plant isolates of *C. jacea*. This is in accordance with the findings of Richards and co-workers [10] studying effective pollen dispersal in the dioecious *Silene alba*. Hence, levels of gene flow by pollen to remote isolated plants should be higher when source populations of insect-pollinated plant species restricted to restored habitats are large but more restricted when source populations are relatively small.

### Conclusions and implications

This study strongly suggests effective pollen dispersal from source populations of *Centaurea jacea* in restored meadows over at least 200 m, even when “recipient populations” consist of single-plant isolates of *C. jacea*. This implies genetic connectedness of most populations of *C. jacea* in restored meadows separated by less than 200 m. Using this species as a model plant, we suggest that other species restricted to restored areas and similar with respect to attractiveness to insect pollinators and pollinator community composition may show similar patterns of effective pollen dispersal. Thus, in the agricultural landscape of the Swiss lowlands, consisting of a very small-scaled mosaic of restored and surrounding intensively managed areas separated on average by less than 100 m (M. Albrecht, unpublished data), most plant populations of neighboring restored areas may be connected by gene flow by pollen. However, our results also indicate a relatively high spatio-temporal variability in effective pollen dispersal, which should also be considered by conservation management.

Our findings indicate that a high diversity and abundance of co-flowering plants in the vicinity can increase the chances of isolated plants or small populations to receive pollinators and pollen from source populations, at least for our model species, and probably for similar plant species, indicating that plant diversity can have positive consequences for ecosystem functioning in intensively managed agricultural landscapes with an embedded network of restored meadows. Moreover, we found evidence that effective pollen dispersal occurred over larger distances if source populations in these restored meadows were relatively large, emphasizing the importance of restoring areas sufficiently large to bear large plant populations. Thus, this study emphasizes that incentives aimed at enhancing the connectivity of insect-pollinated plant populations restricted to restored or protected habitats should consider not only distance but also other factors such as population sizes and the diversity and abundance of co-flowering species.

## References

[1] Saunders DA, Hobbs RJ, Margules CR (1991). Biological consequences of ecosystem fragmentation - A review.. Conserv Biol.

[2] Harrison S, Bruna E (1999). Habitat fragmentation and large-scale conservation: what do we know for sure?. Ecography.

[3] Lienert J, Diemer M, Schmid B (2002). Effects of habitat fragmentation on population structure and fitness components of the wetland specialist *Swertia perennis* L. (Gentianaceae).. Basic Appl Ecol.

[4] Paschke M, Abs C, Schmid B (2002). Effects of population size and pollen diversity on reproductive success and offspring size in the narrow endemic *Cochlearia bavarica* (Brassicaceae).. Am J Bot.

[5] Hooftman DAP, Billeter RC, Schmid B, Diemer M (2004). Genetic effects of habitat fragmentation on common species of Swiss fen meadows.. Conserv Biol.

[6] Kolb A (2005). Reduced reproductive success and offspring survival in fragmented populations of the forest herb *Phyteuma spicatum*.. J Ecol.

[7] Wagenius S (2006). Scale dependence of reproductive failure in fragmented *Echinacea* populations.. Ecology.

[8] Fenster CB (1991). Gene flow in *Chamaecrista fasciculata* (Leguminosae). 1. Gene dispersal.. Evolution.

[9] Ennos RA (1994). Estimating the relative rates of pollen and seed migration among plant populations.. Heredity.

[10] Richards CM, Church S, McCauley DE (1999). The influence of population size and isolation on gene flow by pollen in *Silene alba*.. Evolution.

[11] Ghazoul J (2005). Pollen and seed dispersal among dispersed plants.. Biol Rev.

[12] Schmitt J (1980). Pollinator foraging behavior and gene dispersal in *Senecio* (Compositae).. Evolution.

[13] Kwak MM, Velterop O, VanAndel J (1998). Pollen and gene flow in fragmented habitats.. Appl Veg Sci.

[14] de Jong TJ, Batenburg JC, Klinkhamer PGL (2005). Distance-dependent pollen limitation of seed set in some insect-pollinated dioecious plants.. Acta Oecol.

[15] Klinkhamer PGL, de Jong TJ (1990). Effects of plant size, plant density and sex differential nectar reward on pollinator visitation in the protandrous *Echium vulgare* (Boraginaceae).. Oikos.

[16] Campbell DR (1985). Pollen and gene dispersal - The influences of competition for pollination.. Evolution.

[17] Duncan DH, Nicotra AB, Wood JT, Cunningham SA (2004). Plant isolation reduces outcross pollen receipt in a partially self-compatible herb.. J Ecol.

[18] Moeller DA (2004). Facilitative interactions among plants via shared pollinators.. Ecology.

[19] Ghazoul J (2006). Floral diversity and the facilitation of pollination.. J Ecol.

[20] Waser NM, Jones CE, Little RJ (1983). Competition for pollination and floral character differences among sympatric plant species: a review of evidence.. Handbook of experimental pollination biology.

[21] Schulke B, Waser NM (2001). Long-distance pollinator flights and pollen dispersal between populations of *Delphinium nuttallianum*.. Oecologia.

[22] Baur B (1997). Ökologischer Ausgleich und Biodiversität.

[23] FBS (2004). Biodiversiät in der Schweiz.. Zustand, Erhaltung, Perspektiven.

[24] Swetnam RD, Mountford JO, Manchester SJ, Broughton RK (2004). Agri-environmental schemes: their role in reversing floral decline in the Brue floodplain, Somerset, UK.. J Environ Manage.

[25] OECD (2003b). Agricultural policies in OECD countries: monitoring and evaluation.

[26] Herzog F, Dreier S, Hofer G, Marfurt C, Schupbach B (2005). Effect of ecological compensation areas on floristic and breeding bird diversity in Swiss agricultural landscapes.. Agric Ecosyst Environ.

[27] BLW (2004). Agrarbericht.

[28] Kleijn D, Berendse F, Smit R, Gilissen N (2001). Agri-environment schemes do not effectively protect biodiversity in Dutch agricultural landscapes.. Nature.

[29] Fischer M, van Kleunen M, Schmid B (2000). Genetic Allee effects on performance, plasticity and developmental stability in a clonal plant.. Ecol Lett.

[30] Levin DA, King CE, Dawson PS (1983). Plant parentage: an alternative view of the breeding structure of populations.. Population biology: retrospect and prospect.

[31] Hardy OJ, de Loose M, Vekemans X, Meerts P (2001). Allozyme segregation and inter-cytotype reproductive barriers in the polyploid complex *Centaurea jacea*.. Heredity.

[32] Gardou C (1972). Recherches biosystématiques sur la Section Jacea Cass. et quelques sections voisines du genre Centaurea L. en France et dans les régions limitrophes.. Feddes Repertorium.

[33] Schweiger O, Maelfait JP, Van Wingerden W, Hendrickx F, Billeter R (2005). Quantifying the impact of environmental factors on arthropod communities in agricultural landscapes across organizational levels and spatial scales.. J Appl Ecol.

[34] Crawley MJ (2007). Statistics: an introduction using R.

[35] Vanhoenacker D, Agren J, Ehrlen J (2006). Spatio-temporal variation in pollen limitation and reproductive success of two scape morphs in *Primula farinosa*.. New Phytol.

[36] Petanidou T, Kallimanis AS, Tzanopoulos J, Sgardelis SP, Pantis JD (2008). Long-term observation of a pollination network: fluctuation in species and interactions, relative invariance of network structure and implications for estimates of specialization.. Ecol Lett.

[37] Austerlitz F, Dick CW, Dutech C, Klein EK, Oddou-Muratorio S (2004). Using genetic markers to estimate the pollen dispersal curve.. Mol Ecol.

[38] Morris FW, Price MV, Waser NM, Thomson JD, Thomson B (1994). Systematic increase in pollen carryover and its consequences for geitonogamy in plant populations.. Oikos.

[39] Levin DA, Kerster HW (1968). Local gene dispersal in *Phlox*.. Evolution.

[40] Campbell DR, Waser NM (1989). Variation in pollen flow within and among populations of *Ipomopsis aggregata*.. Evolution.

[41] Widen B, Widen M (1990). Pollen limitation and distance-dependent fecundity in females of the clonal gynodioecious herb *Glechoma hederacea* (Lamiaceae).. Oecologia.

[42] Karron JD, Thumser NN, Tucker R, Hessenauer AJ (1995). The influence of population density on outcrossing rates in *Mimulus ringens*.. Heredity.

[43] Groom MJ (1998). Allee effects limit population viability of an annual plant.. Am Nat.

[44] Lamont BB, Klinkhamer PGL, Witkowski ETF (1993). Population fragmentation may reduce fertility to zero in *Banksia goodii* - A demonstration of the Allee effect.. Oecologia.

[45] Ohashi K, Yahara T (1999). How long to stay on, and how often to visit a flowering plant? - a model for foraging strategy when floral displays vary in size.. Oikos.

[46] Rusterholz HP, Erhardt A (1998). Effects of elevated CO_2_ on flowering phenology and nectar production of nectar plants important for butterflies of calcareous grasslands.. Oecologia.

[47] Klein EK, Lavigne C, Picault H, Michel R, Gouyon PH (2006). Pollen dispersal of oilseed rape: estimation of the dispersal function and effects of field dimension.. J Appl Ecol.

[48] Osborne JL, Clark SJ, Morris RJ, Williams IH, Riley JR (1999). A landscape-scale study of bumble bee foraging range and constancy, using harmonic radar.. JAppl Ecol.

[49] Greenleaf SS, Williams NM, Winfree R, Kremen C (2007). Bee foraging ranges and their relationship to body size.. Oecologia.

[50] Kwak MM, van den Brand C, Kremer P, Boerrichter E (1991). Visitation, flight distances and seed set in populations of the rare species *Phyteuma nigrum* (Campanulaceae).. Acta Hortic.

